# Burden of herpes zoster requiring hospitalization in Spain during a seven-year period (1998–2004)

**DOI:** 10.1186/1471-2334-9-55

**Published:** 2009-05-07

**Authors:** Angel Gil, Ruth Gil, Alejendro Alvaro, María San Martín, Antonio González

**Affiliations:** 1 Department of Health Sciences, Rey Juan Carlos University, Avda de Atenas s/n, 28922 Alcorcón, Madrid, Spain; 2 Medical Department, Sanofi Pasteur MSD, P° de la Castellana 141, 28046 Madrid, Spain

## Abstract

**Background:**

A thorough epidemiological surveillance and a good understanding of the burden of diseases associated to VZV are crucial to asses any potential impact of a prevention strategy. A population-based retrospective epidemiological study to estimate the burden of herpes zoster requiring hospitalization in Spain was conducted.

**Methods:**

This study was conducted by using data from the national surveillance system for hospital data, Conjunto Mínimo Básico de Datos (CMBD). Records of all patients admitted to hospital with a diagnosis of herpes zoster (ICD-9-MC codes 053.0–053.9) during a 7-year period (1998–2004) were selected.

**Results:**

A total of 23,584 hospitalizations with a primary or secondary diagnosis of herpes zoster in patients ≥ 30 years of age were identified during the study period. Annually there were 13.4 hospitalizations for herpes zoster per 100,000 population in patients ≥ 30 years of age. The rate increases with age reaching a maximum in persons ≥ 80 years of age (54.3 admissions per 100,000 population >80 years of age). The mean cost of a hospitalization for herpes zoster in adult patients was 3,720 €. The estimated annual cost of hospitalizations for herpes zoster in patients ≥ 30 years of age in Spain was 12,731,954 €.

**Conclusion:**

Herpes zoster imposes an important burden of hospitalizations and result in large cost expenses to the Spanish National Health System, especially in population older than 50 years of age

## Background

It is estimated that the 15% of the population will suffer from herpes zoster (HZ) at least once in their lifetime [1]. Complications of herpes zoster occurs in 13–26% of the cases [2,3]. Post-herpetic neuralgia (PHN) is the most common and debilitating [4,5], affecting 15 to 40% of cases. [3,6]. The incidence and severity of herpes zoster and postherpetic neuralgia increase with age due to the depletion of cell-mediated immunity to Varicella-Zoster Virus (VZV). Complications are associated with excess of morbidity, but also are thought to be associated with an increase of the costs for the health care system.

An effective and safe, live attenuated varicella-zoster virus vaccine has proved to reduce the burden of illness due to herpes zoster among people 60 years of age or older, as well as the morbidity from post-herpetic neuralgia among older adults [7]. A thorough epidemiological surveillance and a good understanding of the burden of diseases associated to VZV are crucial to asses any potential impact of a prevention strategy.

Hospital discharge databases provide a complete record of all hospitalizations and are not subject to under-diagnosis and deficiencies in reporting that usually limit surveillance systems of outpatient diseases. Additionally, hospitalization databases measure the most severe part of the disease spectrum, herpes zoster in this case [8].

The aim of this study was to estimate the burden of hospital admissions for herpes zoster in persons ≥ 30 years of age, in Spain during a 7-years period (1998–2004).

## Methods

A retrospective study was conducted by using discharge information obtained from the national surveillance system for hospital data maintained by the Ministry of Health, Conjunto Mínimo Básico de Datos (CMBD). This database records all hospital admissions, with an estimated coverage of 97.7% in public hospitals and 25% in private clinics [9,10]. However, private hospitals represent a small proportion of all hospital admissions, as public healthcare insurance is covering almost 100% of the Spanish population [11]. Diagnosis is codified according the Spanish version of the International Classification of Diseases, 9^th ^Revision, Clinical Modification (ICD-9-CM) [12]. Hospital discharge data have been previously shown to be an important source of information for assessing the burden of diseases such as varicella, rotavirus or pneumonia and different types of cancer [13-17]

All hospital discharges with a primary or secondary diagnosis of herpes zoster (ICD-9-MC codes 053.0–053.9) during a 7-year period (1998–2004), were obtained. For each case data were gathered on age, Region of hospitalization, first and secondary diagnosis, type of discharge (death, recovery, other) and length of hospital stay. Costs related to hospitalization were estimated by using the Diagnosis Related Groups (DRG) system, which classifies hospitalizations into groups that are expected to generate similar use of hospital resources. Classification is based on diagnoses, procedures, age, presence of complications and co-morbidities [18]. Each group has similar weight in hospital costs and can be apply to each related patient. GRDs calculations are made by 3 M with Core Grouping System Software [19]

### Statistical methods

The unit of analysis was the hospital admission. The average annual number of hospitalizations for herpes zoster (ICD-9-CM codes 053, 053.0–053.9) in patients ≥ 30 years of age, annual hospitalization rate (annual number of hospital admissions per 100,000 population), average length of hospital stay (ALOS), mortality rate (annual number of deaths at hospital per 100,000 population) and case-fatality rate (annual number of deaths at hospital/annual number of hospital admissions; %) were calculated. The Spanish population obtained from the 1998–2004 census projection and adjusted to the 97.7% of the population covered by hospitals included in the CMBD was used as denominator. It was assumed that the distribution by age of the population covered by public hospitals was equal to the distribution of the general population. The age-standardized hospitalization rates were calculated for the different Autonomous Regions by direct standardization. The standard reference population of the EU was used for this calculation. The frequency of co-morbidities and complications related to herpes zoster was assessed by studying the distribution of the co-existent and subsequent codes to HZ, respectively. The cost of these hospitalizations for the health care system was calculated by considering the diagnosis-related groups (DRGs), the total cost and the number of discharges. ANOVA was used for multiple comparisons. The *post hoc *Bonferroni correction was used to adjust statistical significance for multiple comparisons. The individual cost of a hospitalization was calculated by dividing the total annual cost (all DRG costs per year) by the total number of admissions per year.

Cases of herpes zoster were stratified by age group: 30–49 years; 50–59 years; 60–69 years; 70–79 years; ≥ 80 years. Data were processed and analyzed using SPSS software for personal computers (version 14.0; Chicago, Ill, USA). The study was approved by the Research Ethics Committee of the Hospital 12 de Octubre (Madrid).

## Results

A total of 23,584 hospital discharges with a primary or secondary diagnosis of herpes zoster were identified during the 7-years study period (an annual average number of 3,369 admissions). The mean age (SD) of these patients was 68.3 (15.5) years of age, respectively. Fifty-two percent of the patients were men and more than 85% of these hospitalizations were in population older than 50 years of age (Table 1). The annual hospitalization rate for herpes zoster was of 13.4 hospital admissions per 100,000 population ≥ 30 years of age. This rate increases with age reaching a maximum in population ≥ 80 years of age (54.3 admissions per 100,000 population) (Table 1).

**Table 1:** Hospitalizations for herpes zoster per group of age

Age (years)	Cases	Hospitalization rates (cases per 100,000 (IC95%))	Average length of stay (days)(SD)	Average costs	Total annual costs (1999–2004)
**30–49**	3449	4.13 (3.76–4.49)	12.45 (16.38)	3,983.84 €	1,967,352€
**50–59**	2348	7.44 (7.14–7.75)	13.00 (16.41)	4,078.98 €	1,384,135€
**60–69**	4468	16.56 (16.07–17.04)	13.49 (16.52)	3,796.48 €	2,451,261€
**70–79**	7362	32.87 (32.12–33.62)	12.90 (13.57)	3,616.04 €	3,869,164€
**>80**	5957	54.33 (52.95–55.70)	12.59 (12.36)	3,500.53 €	3,060,044€

**Total**	23584	13.44 (13.27–13.61)	12.88 (14.62)	3,719.53 €	12,731,955€

**Figure 1:**
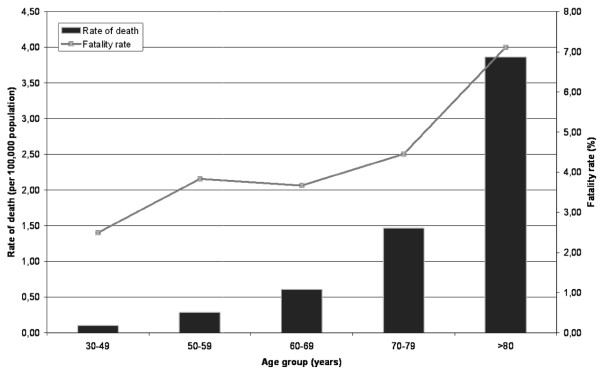
**Rate of death and fatality rate in patients hospitalized with herpes zoster by group of age in Spain (1998–2004)**.

A total of 1,092 deaths occurred among patients hospitalized for herpes zoster during the study period (an average of 156 deaths per year). The case-fatality rate was 4.6% during the study period, being of 7.2% among patients ≥ 80 years old (Figure 1). The mortality rate was 0.6 per 100,000 population, being of 3.9 per 100,000 population among patients ≥ 80 years old (Figure 1). The average length of stay in hospital was 12.9 days (SD 14.6) (Table 1).

**Figure 2:**
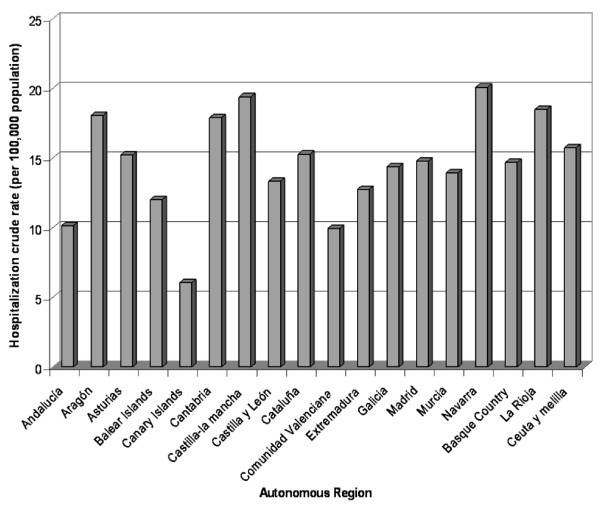
**Hospitalization crude rate due to herpes zoster in Spain (1998–2004)**.

Figure 2 shows the average annual hospitalization rate for herpes zoster in the seventeen Autonomous Regions of Spain. Navarra has the highest hospitalization rate (20.1 per 100,000 population) and Canary Islands the lowest (6.1 per 100,000 population). The average length of hospital stay ranged from 10 days in Murcia to 20 days in Canary Islands.

Herpes zoster code was the first listed diagnosis in 27% of the discharges. In the other 73%, the primary cause of hospitalization (first listed diagnosis) was mainly respiratory diseases (24%) and cardiovascular diseases (19%). When analysing first position diagnosis code, a 3.62 hospitalization rate (per 100,000 population) was found, with a mean age of 68 years old and a mortality rate of 0.06 deaths per 100,000 population. The case-fatality rate was 1.68% and the hospitalization length of stay was 9.23 (SD 9.26).

Twenty-five percent of patients showed to have one or more diagnosis of co-morbid illnesses: leukaemia, other malignancies and HIV were the most frequent (9.6%, 8.4% and 6.5%, respectively), with HIV being the most frequent comorbidity (40%) in the 30–49 year-old patients.

Complications related with herpes zoster were present in 45% of admissions and neurological complications (28.6%) were the most frequently documented conditions in all age groups.

The mean cost of a herpes zoster related hospitalization was € 3,720. This figure was relatively stable among groups of age (Table 1). Therefore, hospitalizations for herpes zoster generate an annual expenditure in Spain of € 12,731,954. When studying separately primary diagnose code, the mean cost per HZ episode was € 3,200.

## Discussion

Analysis of population-based hospital discharge data is a feasible, simple and sensitive way to monitor the occurrence of some diseases and their epidemiology. However, hospital discharge data have some limitations, including some potential biases associated to coding or the fact that a hospital discharge record represents a single hospital admission, and so a single patient admitted more than once will have multiple records [20]. In addition, it was not possible to determine whether herpes zoster was the main cause of the hospitalization. Other limitations are the lack of data on disease severity, medication or use of procedures, such as mechanical respiration, or intensive care stay, all of which are important determinant of hospitalizations usage and costs. [21] However, an outpatient study of herpes zoster in which case finding was based on diagnostic codes, including ICD-9 codes, found an 89%–96% positive predictive value of a diagnostic code of herpes zoster. [22]

More than 23,584 hospitalizations for herpes zoster occurred during the 1998–2004 period, which means an annual hospitalization rate of 13.4 per 100,000 population ≥ 30 years of age for herpes zoster. Our figures are consistent with those found in other studies in USA and Europe using population-based data including general practitioner sentinel surveillance, hospitalisation data, and death certificates. The existing data on the incidence of hospitalization for herpes zoster in Spain is 8.4 per 100,000 population [13]. Studies in the United States and England and Wales show an incidence of herpes zoster-related hospitalizations of 16.1 and 4.4 per 100,000 population, respectively.[8,23] Mainly due to differences in the Health National Systems and criteria for hospitalization of adult patients.

Age-specific rate of hospitalizations increased sharply with age, especially after the age of 50 years, reaching 54.3 hospitalizations per 100,000 persons at ≥ 80 years of age. This finding matches with the pattern of incidence of herpes zoster described in developed countries, where most of the burden of severe zoster occurs in adults ≥ 50 years of age [24,25].

There were some differences among regions in relation to the rate of hospitalizations. These differences may be attributed to variability in the reporting system and coding, and also to factors related to the patient condition (mainly age and comorbidity) and the health system (availability of hospital beds per 100,000 population, clinical procedures). The lower hospitalization rate in the Canary Islands could be explained by its special geographic characteristics, as they are in a tropical area, where the VZV seroprevalence is possibly lower than in temperate areas.

Neurological conditions were the most frequent complication in patients hospitalized for herpes zoster. This has been previously shown in a study carried out in Finland [26]. Persons with underlying comorbidity, such as leukaemia or HIV have an increased risk of presenting zoster-related complications. Anyway, immunocompromised patients will not be prevented by vaccination with the live attenuated varicella-zoster virus vaccine, such admissions might be minimally or non modified by a vaccine-based prevention strategy.

Hospitalization rate, mortality rate and case-fatality rate found for any diagnose position were four, ten and three-fold those for HZ as first diagnosis position, respectively. The hospitalization length of stay was 4 days shorter. This indicates the importance of underlying co-morbidities, that can increase the severity of a herpes zoster episode. The cost for a primary diagnose of HZ was € 520 cheaper.

The estimated annual cost of hospitalizations for herpes zoster in adults in Spain will be of 12.7 million €. This figure will be higher when indirect costs are also considered.

## Conclusion

Herpes zoster is a costly disease that disproportionately affects the elderly. It imposes an important burden of hospitalizations and result in large cost expenses to the Spanish National Health System, especially in population older than 50 years of age

## Competing interests

MSM and AG are employees of Sanofi Pasteur MSD, Madrid, Spain.

## Authors' contributions

AG: study design and its coordination, RG draft the manuscript and review of statistics, AA: statistical analysis, MSM study design, draft the manuscript and study coordination, AG: study design, all the authors have reviewed and approved the final manuscript.

## Pre-publication history

The pre-publication history for this paper can be accessed here:

http://www.biomedcentral.com/1471-2334/9/55/prepub
